# Questioning Sources and Cardiovascular Effects of Nickel

**DOI:** 10.1289/ehp.115-a292

**Published:** 2007-06

**Authors:** Michael D. Dutton

**Affiliations:** CVRD Inco Limited, Toronto, Ontario, Canada, E-mail: mdutton@inco.com

Lippmann et al. (2006) attempted to identify subtle deleterious effects in fine airborne particulate matter (FPM), which is laudable. Nevertheless, the authors’ claim (Lippmann et al. 2006) that on 14 of 103 days studied in the fall of 2004, concentrated air pollutants (CAPs) at Tuxedo, New York [near New York City (NYC)], contained “greatly elevated concentrations of nickel attributable to the Ni smelter at Sudbury, Ontario” is unsubstantiated. The Ni concentrations to which they referred (174 ng/m^3^) on these 14 days were concentrated 10-fold from ambient air. In other words, the Ni in ambient air at Tuxedo was actually only 17.4 ng/m^3^, the same as NYC (19 ng/m^3^).

Lippmann et al. (2006) assigned the “elevated” Ni to Inco’s 381 m stack in Sudbury based on back trajectory analyses. The authors failed to account for vertical components of air parcel movement; also, by singling out one specific trajectory from the “NW wind” days, they implied much more accuracy to the back trajectories than is justified. Because meteorological data are available at 3-hr intervals, three back trajectories could be developed for each daily exposure period. Such back trajectories using the internet-based HYSPLIT model (National Oceanic and Atmospheric Administration 2006) indicate that for the 14 NW wind days, elevated Ni in CAPs on those days was more likely due to sources other than the Sudbury stack > 800 km distant. Furthermore, Inco’s stack emissions are characteristic and distinct from the CAPs composition reported by Lippmann et al. (2006). Concentrations of aluminum, chromium, and iron are all 100-fold less than the concentration of Ni in Inco’s emissions, whereas in CAPs on the NW wind days, the ratios of Cr:Ni and Fe:Ni, as well as those of Al:Ni and V:Ni, are similar to those of New Jersey air (Reinfelder et al. 2004). The Ni in ambient air at Tuxedo, even on the 14 NW wind days, could be easily assigned to sources surrounding NYC. Given that Tuxedo is near NYC, it should be no surprise that local sources could be large contributors to Ni in ambient FPM in Tuxedo. Although the atmospheric Ni emissions from the Sudbury stack can be transboundary and have been significant historically, the incremental contribution of Ni from Sudbury to ambient air at Tuxedo in 2004 would have been dwarfed relative to local sources. Significant reductions in emissions have been made at Inco’s Sudbury operations, and air emissions from the Inco stack will diminish further as new pollution control measures are implemented.

Lippmann et al. (2006) presented two lines of evidence that Ni is the major cause of cardiovascular effects of FPM. First, exposures of ApoE^−/−^ mice to CAPs led them to conclude that unusually high heart rate (HR) occurred in response to elevated Ni on the NW wind days. Although the largest sustained apparent difference in HR occurred through most of December, only three NW wind days occurred in that month. Other changes in HR and heart rate variability (HRV) are either due to changes in control animals or occurred when there were no elevated Ni concentrations [see Figure 4 of Lippmann et al. (2006)]. There appears to be an error in the key of Lippmann et al.’s Figure 4 compared with the original manuscript published online: the solid lines now denote filtered air (control) instead of CAPs, and the dashed line indicates CAPs instead of control data. Given that the authors referred to the elevated HR in exposed mice, the key in the print version must be incorrect.

Considering that 2-year inhalation exposures of rodents to nickel sulfate at levels 600 times higher than those used by Lippmann et al. (2006) were without effect on mortality, the relevance of the “subtle” changes in HR and HRV requires further thought. Second, the authors “wondered if Ni may have been responsible for the notably high daily mortality” in NYC. Although it is true that NYC has a cardiovascular and respiratory (CVR) mortality coefficient that is above the national average, 34 of the 90 National Mortality and Morbidity Air Pollution Study (NMMAPS) cities have CVR mortality coefficients greater than those of NYC. Furthermore, there is no statistical relationship between NMMAPS CVR mortality rates and Ni emission rates (U.S. Environmental Protection Agency 2002).

The conclusions of Lippmann et al. (2006) contrast with the recent assessments of the Agency for Toxic Substances and Disease Registry (ATSDR 2005) and the European Union (European Chemicals Bureau 2005) that did not identify human cardiovascular risk factors for Ni. Historical monitoring within the Ni industry identified the link between high occupational exposure of certain Ni species and respiratory cancer, but no such cardiovascular risk factors have been identified after decades of occupational health monitoring.

Further research to evaluate the impacts of the constituents of FPM on cardiovascular health is justified and should continue. Nevertheless, researchers with appropriate expertise work should cooperatively in studies such as these. In this instance, Lippmann et al. (2006) would have benefitted greatly from the inclusion of atmospheric scientists on their research team.
